# Cytokines induce effector T-helper cells during invasive aspergillosis; what we have learned about T-helper cells?

**DOI:** 10.3389/fmicb.2015.00429

**Published:** 2015-05-11

**Authors:** Raman Thakur, Rajesh Anand, Shraddha Tiwari, Agam P. Singh, Bhupendra N. Tiwary, Jata Shankar

**Affiliations:** ^1^Department of Biotechnology and Bioinformatics, Jaypee University of Information Technology, Solan, India; ^2^Infectious Diseases Laboratory, National Institute of Immunology, New Delhi, India; ^3^Department of Biotechnology, Guru Ghasidas Vishwavidyalaya, Bilaspur, India

**Keywords:** cytokines, T-helper cells, dendritic cells, *Aspergillus*, antigen presenting cells, invasive aspergillosis

## Abstract

Invasive aspergillosis caused by *Aspergillus* species (*Aspergillus fumigatus*, *A. flavus*, and *A. terreus*) is life-threatening infections in immunocompromised patients. Understanding the innate and adaptive immune response particularly T-helper cells (T_H_-cells) against these *Aspergillus* species and how the different sub-set of T_H_-cells are regulated by differentiating cytokines at primary target organ site like lung, kidney and brain is of great significance to human health. This review focuses on presentation of *Aspergillus* through Antigen presenting cells (APCs) to the naive CD4^+^ T-cells in the host. The production of differentiating/effector cytokines that activate following T_H_-cells, e.g., T_H_1, T_H_2, T_H_9, and T_H_17 has been reported in association or alone in allergic or invasive aspergillosis. Chemokines (CXCL1, CXCL2, CCL1, and CCL20) and their receptors associated to these T_H_-cells have also been observed in invasive aspergillosis. Thus, further study of these T_H_-cells in invasive aspergillosis and other elements of adaptive immune response with *Aspergillus* species are required in order to have a better understanding of host response for safer and effective therapeutic outcome.

## Introduction

Fungi are the most common microorganisms and have clinical importance. Few of them are pathogenic or opportunistic pathogen and results in morbidity and mortality to human beings. There is a rise in opportunistic fungal infections in recent years due to increased incidence of immunocompromised host (Chamilos et al., 2006; Romani, 2008). After *Candida albicans*, the leading causes of fungal infections in immunocompromised individuals are from *Aspergillus* species. *Aspergillus* is one of the most ubiquitous medically important opportunistic fungi (Weaver et al., 2007). The genus *Aspergillus*, contains about 40 species that can cause infection (Verweij and Brandt, 2007), among them *A. fumigatus*, *A. flavus*, and *A. terreus* are the leading cause of invasive Aspergillosis in immunocompromised individuals. These species produce conidia at a concentration of around 1–100 conidia per m^3^ (Barnes and Marr, 2006). Human routinely inhale hundreds of these conidia per day, despite these exposure to *Aspergillus* conidia, human do not develop any disease due to the clearance of conidia from lung by innate immunity especially phagocytic cells (Chamilos et al., 2006; Romani, 2008). However, due to rise in immunocompromised host, e.g., patients receiving organ transplant, immunosuppressive therapy for autoimmune or neoplastic disease and HIV patients, inhaled conidia if not cleared in these host, colonization of *Aspergillus* occurs (Stevens et al., 2000). The adaptive immune response in human responsible for conidia clearance is not well understood in immunocompetent host as well as where conidia colonize in immunocompromised host. It is worth to note that secondary metabolites (e.g., Gliotoxins, Aflatoxins) excreted by *Aspergillus* especially have been recognized to modulate immunological responses (Shankar, 2013). Thus, we reviewed recent advances made in immune responses against *Aspergillus* species in mice model studies and clinical aspergillosis patients. *A. fumigatus* is the prominent species, which cause 90% of Aspergillosis followed by *A. flavus* and *A. terreus*. Studies have showed that *Aspergillus* species associated with infection after hematopoietic stem cell transplantation include *A. fumigatus* with 56% followed by *A. flavus* (18.7%) and *A. terreus* (16%) (Steinbach et al., 2004; Morgan et al., 2005). The involvement of *Aspergillus* infection in pulmonary tuberculosis and in asthmatic patients has been reported by Denning et al. It has been estimated annually at least 372,385 patients developed chronic pulmonary aspergillosis worldwide following treated pulmonary tuberculosis (Denning et al., 2011). Similarly, around 4,837,000 patients develop Allergic bronchopulmonary aspergillosis out of 193 million adults with active asthma (Denning et al., 2013). However, in a recent study of Indian population by Agarwal et al. (2014) the estimated ABPA burden was 1.38 million out of 27.6 million adults with asthma. This review touches different aspect of antifungal immunity against aspergillosis that include antigen presenting cells (APCs), dendritic cells (DCs), fungal pattern recognition receptors (PRRs), T_H_-cells with their subsets profile during infection associated to *Aspergillus* species at different site of infection, e.g., lung, kidney, and brain.

## Recognition of *Aspergillus* by the Host

### Presentation of Pathogen via Soluble Receptors and Cell Bound Receptors

After the inhalation of *A. fumigatus* conidia, they are entrapped by the lung alveoli and if they are not efficiently cleared from lung, they germinate and establish lung infection termed invasive pulmonary aspergillosis and it may also disseminates to other organs if not treated (Park and Mehrad, 2009). The recognition of *A. fumigatus* conidia and hyphae occurs by PRRs those include soluble receptors and cell-bound receptors. Conidial germination starts with hydrophobic layer degradation and exposure of inner cell wall components mainly polysaccharides, which includes chitin, β-glucan, mannan, and galactomannan. These are termed as pathogen associated molecular patterns (PAMP), are recognized by PRRs (Netea et al., 2006; Inoue and Shinohara, 2014). PRRs soluble receptor such as pulmonary collectins, family of C-type lectins, pentraxin-3, pulmonary surfactant proteins-A and D have been reported in aspergillosis. Further, the cell-bound receptors in association with aspergillosis include Toll like receptor-2 (TLR), TLR-4 and TLR-9, which potentially induce the production of pro-inflammatory cytokines and reactive oxygen species through MyD88 signaling pathway (Willment and Brown, 2008).

## Antigen Presenting Cells and T-cell Differentiation

### Antigen Presenting Cells Triggers Cytokines Production

The activation of the innate immunity through PRRs present on the APCs that regulate the development of T-cell. APCs express wide-array of PRRs that provides the link between adaptive and innate immunity (Park and Mehrad, 2009). APCs, dominantly DCs, are responsible for antigen monitoring and then shaping T-cell response by secreting cytokines and chemokines. DCs express PRRs on their cell surface and endosomal compartments, which serve to recognize PAMPs. After interaction with DCs, naive T-cells are activated. The activation of T-cell response is regulated by the cytokines milieu predominantly framed by DCs (Akdis et al., 2011). Chemokines secreted by DCs recruit the phagocytic cells to infected areas to clear the *Aspergillus* components. APC cells, e.g., monocytes differentiate into distinct sub-populations of CD14^+^ and CD16^+^ cells after *A*. *fumigatus* conidia infection (Serbina et al., 2009). Monocytes interact with *Aspergillus* antigens resulting in maturation of monocytes into macrophages or DCs. Macrophages and DCs interact with antigens and secrete effector cytokines (Osugi et al., 2002; Ramirez-Ortiz and Means, 2012). Major sub-populations of DCs are myeloid DCs, plasmacytoid DC (pDCs) and monocyte-derived DCs (Bozza et al., 2002; Osugi et al., 2002). pDC recognize the nucleic acids from *A. fumigatus* via TLR-9 and lead to resistance to *A. fumigatus* infection in mice (Ramirez-Ortiz et al., 2008). Further, monocytes migrate toward the lung to differentiate into either DCs or alveolar macrophages during invasive aspergillosis (Cramer et al., 2011; Morton et al., 2012). Monocytes express different chemokines receptor predominantly CCR2, which help in migration of monocytes from bone marrow toward lung in response to *A. fumigatus* infection (Serbina et al., 2009). It has been shown that monocytes expressing CCR2 in the lung involved in conidial uptake and killing (Espinosa et al., 2014). Furthermore, alveolar macrophages induce APCs to release IL -1β in pulmonary invasive infection (Park and Mehrad, 2009). IL-18 has also been observed in lung during invasive aspergillosis mice model (Akdis et al., 2011). Recently, it has been observed that *A. fumigatus* pulmonary challenge induces expression of the inflammasome-dependent cytokines IL-1β and IL-18 within the first 12 h, while IL-1α expression continually increases over at least the first 48 h (Caffrey et al., 2015). Moretti et al. (2014) showed in a pulmonary invasive aspergillosis model that mice injected with IL-37 prior to *A*. *fumigatus* infection has significant reduction in IL-β production and recruitment of neutrophils and resulted in diminution in lung inflammation and damage. The anti-inflammatory activity of IL-37 has been observed as an inhibitor of the innate response. Thus, cytokines play a vital role in modulation of immune response and coordinate the innate as well as adaptive responses. APCs secrete cytokines that act on naïve T-cells leading to the differentiation of naïve T-cells. These differentiated T-cells further secrete effector cytokines and regulate the function of T_H_-cells. The profile of cytokine depends on the type of *Aspergillus* antigens, route of infection, immunological status of the host and cytokines milieu present during the interaction (Romani, 2008; Chai et al., 2010b). CD4^+^ T-cells can be divided into distinct subtypes according to cytokine profile, and they can differentiate to T_H_1, T_H_2 T_H_17, T_H_9, and T-follicular effector cells (Kerzerho et al., 2013; Kara et al., 2014). On the basis of the cytokine profile, these T_H_-cells perform distinct functions. However, it is not clear how T-follicular effector cells respond during *Aspergillus* infection (Wüthrich et al., 2012).

### Cytokines Associated with T_H_1 Type of Response

*Aspergillus fumigatus* challenged intranasally in mice interacts with DCs and alveolar macrophages in the lung. Secretion of T_H_1 associated pro-inflammatory cytokines IL-12, IFN-γ, TNF-α, IL-18 has been observed after the challenge (Chotirmall et al., 2013). Among these, IL-12 is the prominent cytokine released from activated monocytes and macrophages in lung that help in shaping T-cell immune response. IL-12 is a heterodimeric cytokine composed of IL-12p35 and IL-12p40 polypeptides that form the bioactive IL-12p70. The heterodimer binds to the IL-12 receptor composed of IL-12Rβ1 and IL-12Rβ2 chains and signals through STAT-4 (Shao et al., 2005). IL-12 acts on NK cells to promote IFN-γ secretion and differentiate CD4^+^ T-cells into T_H_1-cells, once CD4^+^ cells differentiates to T_H_1-cells, they increase the secretion of IFN-γ, which suppress T_H_17 and T_H_2 response in the lung (Espinosa and Rivera, 2012; Camargo and Husain, 2014). IL-12, hence, is the most important regulator of T_H_1 response during lung infection. IL-12 deficient mice failed to generate a T_H_1 response, leading to increased secretion of IL-4 and IL-10 cytokines, which shifts the immune response toward T_H_2 pathway (Cenci et al., 1998). In *A. fumigatus* induced neutropenic aspergillosis in mice, NK cells can be the primary source of IFN-γ responsible for activating phagocytic cells and direct antifungal effectors cells against *A. fumigatus* (Park et al., 2009). Further, patients with invasive *Candida* and/or *Aspergillus* infections, recombinant treatment of IFN-γ in combination with antifungal drug partially restored immune function (Delsing et al., 2014). In intravenous infection of *A. flavus* mice model studies, lung and brain homogenate showed pro-inflammatory cytokines IL-12 and IFN-γ and relative absence of IL-4, IL-23, and IL-17 suggesting a T_H_1 response (Anand et al., 2013, 2015). *A. terreus* induced invasive aspergillosis showed the presence of IL-1β, IL-6, and reduced level of IL-10 in mice model studies. Although there is activation of T_H_17 type of adaptive immune response through IL-1β but the later is suppressed by T_H_1 cytokines particularly IFN-γ (Vyas, 2011; Lass-Florl, 2012). The T_H_1 response is thus also mounted by *A. terreus* and there is a lack of T_H_2 response in contrast to *A. fumigatus* infection where T_H_2 promoting cytokines are observed.

### Cytokines Associated with T_H_17 Type Response

*Aspergillus fumigatus* mediated infections in lung induce T_H_17 and T_H_1-cells. These cells play an important role in protection and induction of inflammation (Chai et al., 2010b). Activation of T_H_17-cell depends on Dectin-1 signaling pathway. Various studies have suggested that dectin-1 deficient mice entirely activate T_H_1-cells. So Dectin-1 signaling not only serves as a positive factor to promote T_H_17 differentiation but rather act to balance T_H_1 versus T_H_17 differentiation. Activation of the APCs by Dectin-1, release the proinflammatory cytokines IL-1β, IL-6, IL-23, and IL-22 which differentiates CD4^+^ T-cells to T_H_17-cells, which further secretes IL-17A and IL-17F cytokines and maintain T_H_17 response (Werner et al., 2011). IL-23 is a member of IL-12 family, produced by phagocytic cells, macrophages and activated DCs in lung. IL-23 contains two subunits IL-12p40 and IL-23p19 and it binds to heterodimeric receptors IL-12Rβ1, expressed by activated T-cells (Zelante et al., 2007). IL-6 is another important cytokine involved in regulation of T_H_17 response. IL-6 is a multifunctional cytokine, promote T_H_17-cells differentiation, inflammation and acute response (Akdis et al., 2011). During T_H_17 differentiation, human naïve T-cells are exposed to IL-1β, IL-6, and IL-23 (Zelante et al., 2009; Gresnigt and van de Veerdonk, 2014). T_H_17 promoting cytokine IL-17 binds to IL-17RA and IL-17RC receptors expressed in lung cells, like fibroblast, epithelial cells and T-cells. After release of IL-17 from T_H_17-cells, it activates the neutrophils migration toward infected area and increases inflammation (Wilson et al., 2007). In *A. flavus* and *A. terreus*, the role of T_H_17-cells during lung infection is yet to be established.

### Cytokines Associated with T_H_2 Type of Response

*Aspergillus fumigatus* is associated with both invasive and allergic form of aspergillosis. In case, if conidia are not cleared, they germinate to produce hyphae, which are responsible for invasion in host tissues that leads to inflammation. In a healthy human T-cells response, *A. fumigatus* not only evoke pro-inflammatory type of immune response via T_H_1 and T_H_17-cells but also anti-inflammatory type of immune response mediated by T_H_2-cells (Chaudhary et al., 2010). Immune response initiated by IL-4 and IL-10 inhibits T_H_1 and T_H_17 response and increased secretion of IL-4 and IL-10 inhibits IFN-γ and IL-12 production. T_H_2-cells differentiation depends on IL-4 and IL-10 and after differentiation in to T_H_2 cells, these cells further secretes IL-5 and IL-13 which maintain T_H_2 response. T_H_2 immune response is triggered in acute bronchopulmonary aspergillosis and also in invasive pulmonary infection during some time point of infection. IL-4 and IL-10 deficient mice show lower *A. fumigatus* burden and increased survival rates compared to wild type mouse in invasive pulmonary aspergillosis (Cenci et al., 2000). It has been shown that ESTs (L3 ribosomal protein, L7A ribosomal protein, Histone -H2A) have high sequence similarity with human counter parts suggesting molecular mimicry between human and pathogen protein (Shankar et al., 2004). However, role of these genes in eliciting allergic immune response needs investigations. In *A. flavus* mediated infection, lung homogenate showed the absence of T_H_2 response in a limited cytokine profile study (Anand and Tiwary, 2010). However, T_H_2 type response may get activated in later stages of infection in lung due to rise in IL-4 and IL-10, which suppress the T_H_1 response but consistent expression IFN-γ overcomes T_H_2 response. In addition to T_H_2, the role of T_H_9-cells has been shown during infection with a Virus, bacteria, parasites and fungi. T_H_9 response contributes to allergic inflammation during allergic aspergillosis due to *A. fumigatus* in mice model (Kerzerho et al., 2013). T_H_9-cells develop in the presence of IL-1α and TGF-β along with T_H_2-cells. However, role of T_H_9 and T_H_2 response during invasive aspergillosis remains unclear.

## Is Their Co-evolution of T-helper Cells During Invasive Aspergillosis?

In a immunocompromised mice model studies, repeated exposure of *A. fumigatus* conidia in hosts lead to co-evolution of T_H_1, T_H_2, and T_H_17 response in infected lung (Murdock et al., 2011). They have observed the presence of IFN-γ and IL-17 in infected lung of mice along with T_H_2 response. These mixed responses might be occurring at different time points during progression of infection and leads to either protection or infection. T_H_1 and T_H_17 response probably leads to protection where as T_H_2 response further complicates the disease. T_H_2 response help in evasion of *A*. *fumigatus* from immune cells and further increase the IgE level, which leads to high inflammation at the site of infection. Mice model of ABPA demonstrated the T_H_2 cytokine profile consisting of IL-4, IL-10, and IL-5 (Latge, 1999).

## Interplay of Cytokines; T_H_1 or T_H_2 or T_H_17 Type of Response

**FIGURE 1:**
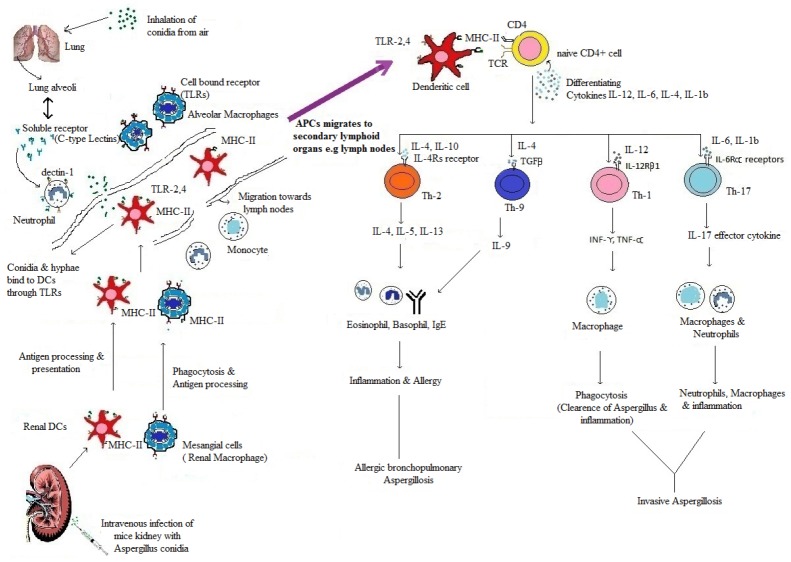
**The recognitions, processing and presentation of *Aspergillus* antigens to naïve CD4^+^ T_H_-cells and Production of effector T_H_-cells.** In infected Lung and Kidney, *Aspergillus* antigens (conidia & germinating conidia) are recognized by PRRs, i.e., soluble (C-type lectins) and cell bound receptors; TLR2, TLR4, and TLR9 (Netea et al., 2006). They are present on antigen presenting cells (DCs and macrophages). After recognition, antigens recognized by APCs, process and present to naïve CD4^+^ T-cells in secondary lymphoid organs (Chai et al., 2010a). After interaction of APCs and naïve CD4^+^ T-cells, differential cytokines release (IL-12, IL-6, IL-4, IL-1β) (Korn et al., 2009; Chai et al., 2010a) act upon CD4^+^ T-cells and differentiate them into effector T_H_-cells. IL- 12 give rise to T_H_1, IL-6, IL-1β give rise to T_H_17 and IL-4 give rise to T_H_2 effector T_H_-cells (Murdock et al., 2011). These effector cells further secrets effector cytokines (IFN-γ, TNF-α, IL-10, IL-5, IL-17, and IL-23) (Zelante et al., 2007) which maintain effector T_H_-cells response. Figure shows summary of development of effector T_H_-cells response during Lung and Kidney infection of *Aspergillus*. The figure is not to the scale.

T_H_-cells response during invasive *Aspergillus* depends on differentiating cytokines. T_H_1 response is activated by differentiating cytokine IL-12 followed by secretion of IFN-γ. Secretion of IFN-γ further stimulates T_H_1 cells, if IFN-γ dominates initially it suppress the other cytokines of T_H_2 and T_H_17, i.e., IL-4 and IL-17 (Harrington et al., 2005). If IL-4 dominates during initial period of *Aspergillus* infection, it suppresses the protective T_H_1 type immune response by inhibiting differential cytokine IL-12 and IFN-γ (Harrington et al., 2005). Recognition of *Aspergillus* antigens by Dectin-1 signaling pathway inhibit the production of IFN-γ and IL-12 receptors suppressing T_H_1, which leads to differentiation of T_H_17-cells and production of IL-17. In this way Dectin-1 signal balances the T_H_1 and T_H_17 response through the regulation of their respective cytokines (Rivera et al., 2011; Figure 1). The development of effective CD4^+^ T_H_-cells response not only depends upon cytokines, but also on chemokines and their receptors. Chemokines help in recruitment of leukocytes, i.e., neutrophils, monocytes and NK cells toward lung during *Aspergillus* infection. These cells express chemokine receptors; neutrophils contain CXCR2 chemokine receptor for ligand CXCL1 and CXCL2, monocytes contain CCR2 and CCR6 receptor for CCL2 and CCL20 ligands, where as NK cells contain CCR2 receptor for CCL2 ligand. So these chemokine ligands attract monocytes, neutrophils and NK cells to clear the *Aspergillus* hyphae during lung infection (Park and Mehrad, 2009). These chemokines receptors are also present on DCs, regulatory T-cells (Tregs) and T_H_-cells and help in their trafficking (Bendall, 2005). In this way, there is an interdependent relationship between chemokines and cytokines that help in evolution of effector T_H_-cells response. CCL17, a chemokine, help in trafficking of DCs, Tregs and T_H_1-cells toward infected area during invasive aspergillosis in response to CCR4 chemokine receptor present on these cells. Further, CCR6 receptor present on DCs and T_H_17-cells help in migration of these cells in response to chemokine CCL20 (Bendall, 2005; Wüthrich et al., 2012).

## Conclusion

Cytokines are important in the development of CD4^+^ T_H_-cells. Understanding of trafficking of CD4^+^ T_H_-cells and their regulation through differentiating/effector cytokines during invasive aspergillosis will be crucial for the targeted immunotherapy. Overall, cytokines and chemokines may serve as prognostic biomarkers that could be followed to assess the effectiveness of treatment response during invasive aspergillosis. Measurement of selected cytokines in the blood samples of aspergillosis patients may be a promising tool for the monitoring of treatment responses. Also, manipulation of cytokine response e. g, IFN-γ or IFN-γ in combination with antifungal drug, IL-37, may be a future avenue for the development of better therapeutic against invasive aspergillosis.

### Conflict of Interest Statement

The authors declare that the research was conducted in the absence of any commercial or financial relationships that could be construed as a potential conflict of interest.
